# TiO_2_ nanomaterial promotes plant growth and disease resistance

**DOI:** 10.1080/15592324.2025.2512943

**Published:** 2025-05-30

**Authors:** Xiaotong Gai, Xiaofeng Xu, Ning Jiang, Dingli Zhang, Yongjun Zhang, YongWn Kim, YuanHu Xuan, Dandan Li

**Affiliations:** a Plant Protection Technology Research, Yunnan Academy of Tobacco Agricultural Sciences, Kunming, China; b College of Plant Protection, Shenyang Agricultural University, Shenyang, China; c Technique Center, Lincang Tobacco Company of Yunnan Lincang, Lincang, China; d Management Department, Small & Medium Business Corporation, Jeonju, South Korea; e State Key Laboratory of Elemento-Organic Chemistry and Department of Plant Protection, National Pesticide Engineering Research Center, Nankai University, Tianjin, China

**Keywords:** TiO_2_, plant growth promoting, disease resistance, nutrient uptake, yield increase

## Abstract

TiO_2_ nanomaterials can promote plant growth and enhance disease resistance. However, the underlying mechanism remains unclear. This study applied TiO_2_ to promote the growth of wheat, soybean, tobacco, cucumber, and corn. Genetic analysis using macro-element transporter rice mutants in rice revealed that growth promotion induced by TiO_2_ was dependent on *potassium transporter* (*AKT1*), *nitrate transporter 1.1B* (*NRT1.1B*), *ammonium transporter 1* (*AMT1*), and *phosphate transporter 8* (*PT8*). TiO_2_ also enhanced chlorophyll accumulation, and growth promotion was inhibited in the chlorophyll biosynthesis rice mutants, *yellow-green leaf 8* (*ygl8*) and *divinyl reductase* (*dvr*), indicating that TiO_2_ promoted growth through chlorophyll biosynthesis. In addition to photosynthesis, TiO_2_ affected light signaling by inhibiting the translocation of Phytochrome B (*PhyB*) from the cytosol to the nucleus, thereby improving resistance to rice sheath blight (ShB). TiO_2_ application also enhanced resistance to wheat stem rust, tobacco wildfire, angular spot disease, and rice ShB by inhibiting the growth of bacterial and fungal pathogens, suggesting that TiO_2_ regulates plant defense signaling and has antibacterial and antifungal effects. Field experiments with wheat, soybeans, and rice confirmed that TiO_2_ treatment significantly increased the crop yield. These findings suggest that TiO_2_ is a promising nanomaterial for the simultaneous enhancement of plant growth and disease resistance.

## 1. Introduction

Nanomaterials (NPs) are utilized in diverse fields, including life sciences, aerospace, electronics, and agriculture, owing to their unique physicochemical properties.^1,2^ In recent years, the development of NPs for agricultural applications, particularly the application of nanopesticides and nanofertilizers for pollution management and sustainable agriculture, has garnered significant research interest.^2–6^ Notably, nanopesticides have been recognized as one of the “Top Ten Chemical Innovations”, highlighting the potential of NPs in eco-friendly agriculture.^7^ TiO_2_ NPs are important functional semiconductor materials that have been widely applied in electronics, chemical production, environmental protection, and other fields.^8–10^ Furthermore, they can generate cytotoxic reactive oxygen species (ROS) upon photoactivation, rendering them valuable for biomedical applications such as antibacterial, antiviral, anticancer, antioxidant, and drug delivery systems.^11^

The silver (Ag)-, titanium (Ti)-, and copper (Cu)-based NPs are commonly employed as nanopesticides owing to their antibacterial or insecticidal properties.^12–14^ Among them, TiO_2_ NPs are considered excellent antibacterial agents owing to their unique photocatalytic properties. TiO_2_ NPs are relatively environmentally friendly and have been adopted safely and extensively for environmental, industrial, and agricultural applications.^11^ In nature, TiO_2_ exists mainly in the form of anatase, rutile, and plate-like titanium.^15^ The physiological effects of TiO_2_ NPs depend on plant species, nanoparticle size, crystal phase, concentration, and exposure time. Studies have shown that TiO_2_ NPs positively affect seed germination and root elongation and are nontoxic to plants, even when titanium is absorbed.^16–19^ TiO_2_ NPs can reduce disease severity, promote plant growth, enhance flower and fruit yields, and increase carotenoid content and enzyme activity.^5,11^ In addition, TiO_2_ NPs are biocompatible and cost-effective fungicides.

Photosynthesis is crucial for plant growth and serves as the foundation for crop yield.^20^ Chlorophyll content is positively correlated with the photosynthetic rate and biomass accumulation.^21^ Studies have indicated that spraying 20 mg/L TiO_2_ NPs on strawberries can increase fruit length, chroma index, and carbohydrate content by 19.8% under drought stress.^22^ Additionally, TiO_2_ NPs can enhance plant height, fresh weight, leaf tissue protection, stomatal opening, and antioxidant system performance in *Medicago sativa* L.^23^ When applied to the leaf surfaces of green vegetable seedlings, TiO_2_ NPs (20 mg/L) significantly increased biomass, total phosphorus, and catalytic enzyme activities.^24^ TiO_2_ NPs can improve plant growth indicators, including fresh biomass and chlorophyll content, by enhancing photosynthetic efficiency. This can be achieved by increasing photosynthetic parameters, such as photosynthetic rate, stomatal conductance, intercellular CO_2_, and transpiration rate, ultimately promoting biomass accumulation. These findings offer promising insights into the utilization of NPs to boost crop yield.^24^

NPs, especially TiO_2_ NPs, have shown great potential in agricultural applications. However, the underlying mechanisms of how TiO_2_ affects plant growth and disease resistance remain not fully understood. Therefore, the aims of this study are as follows:

Firstly, we will explore whether TiO_2_ can promote plant growth through pathways such as nitrogen (N), phosphorus (P), and potassium (K). We will study its influence on nutrient absorption in rice and other crops, aiming to clarify the role of these pathways in TiO_2_-mediated growth promotion. Secondly, we will study the effect of TiO_2_ on the biosynthesis of chlorophyll in rice and explore the relationship between the change of chlorophyll content and the promotion of plant growth. Thirdly, we will evaluate the effect of TiO_2_ in enhancing the resistance of plants to diseases such as wheat rust, rice ShB, tobacco wildfires, and angular spot disease. Fourthly, we will conduct field experiments to provide actual data for the application of TiO_2_ in improving crop productivity. Finally, we will explore the influence of TiO_2_ on photosynthesis and light signals. Given that TiO_2_ may have an impact on the nucleation of Phytochrome B (PhyB), we will study its differential regulatory role in these processes, which will contribute to a better understanding of the overall impact of TiO_2_ on the physiological functions of plants. In conclusion, this study aims to comprehensively evaluate the potential of TiO_2_ as a nanotherapy agent in promoting plant growth and enhancing pathogen resistance, providing valuable insights for its further application in agriculture.

## 2. Materials and methods

### 2.1. Plant growth conditions and inoculation method of Rhizoctonia solani AG1-IA

Rice varieties, including Dongjin (DJ), Zhonghua11 (ZH11), Nipponbare (Nip), ammonium transporter mutant (*amt1*), nitrate transporter mutant (*nrt1.1b*), phosphate transporter mutant (*pt8*), and potassium channel (*akt1*), were cultivated in a greenhouse under controlled conditions (24–30°C, 70% relative humidity) at Nankai University before transplantation. The DJ rice was transferred to MS liquid medium with varying concentrations of TiO_2_ (72 ppb, 36 ppb, 18 ppb and 9 ppb) and cultured for 5 d to measure shoot length and determine the optimal growth-promoting TiO_2_ concentration. Subsequently, ZH11, Nip, *amt1*, *nrt1.1b*, *pt8*, *akt1, SH498*, *gyl8*, *dvr*, and *gs1;1* rice plants were transferred to Murashige and Skoog (MS) liquid medium with or without TiO_2_ and cultured at 28℃, 50% relative humidity, and 12-hour light/dark cycles for 5 d to measure shoot length and calculate growth promotion rates. *Rhizoctonia solani* AG1-IA was grown on Potato Dextrose Agar (PDA) medium at 28℃ for 2 d, and the rice inoculation followed a previously established method.^25^ Lesion analysis was conducted using ImageJ software.

### 2.2. Promoting growth effect of TiO_2_ on different crops

Corn, wheat, soybean, and cucumber were directly seeded in a 3:1 mixture of matrix soil and vermiculite, whereas tobacco was sown after germination. After 3 d of germination in water, tobacco seeds were sown in a soil-vermiculite mixture. They were grown in a glass greenhouse at Nankai University for 7 d, with TiO_2_ at a concentration of 9 ppb applied every 3 d for a total of three times.

### 2.3. Chlorophyll concentration determination

DJ rice seeds were disinfected by soaking in 0.6% H_2_O_2_ for 30 min, washed three times with water, and incubated at 25℃ for germination. After 5 d of bud growth, the seedlings were transferred to liquid MS medium. 10 ml of TiO_2_ with a concentration of 9 ppb were sprayed onto the rice leaves once daily, and the leaves were collected after 7 d of cultivation for chlorophyll content measurement following a previously published method.^26^

### 2.4. Inoculation method of wheat stem rust

*Puccinia graminis* f. sp. *tritici* (*Pgt*) was removed from the −80℃ storage and activated in a 42°C water bath. Wheat was grown in porcelain pots in a glass greenhouse at Nankai University for 7 d, and 0.1% Tween 20 was evenly applied to the wheat leaves. The activated *Pgt* strains were mixed with talc powder at a 1:20 volume ratio and sprayed onto wheat leaves. The plants were kept in darkness at 18–22℃ for 12–14 h and then transferred to a greenhouse under the same temperature conditions. The inoculated leaves were photographed 14 d later. Specific inoculation methods followed previously published protocols.^27^

### 2.5. Inoculation method of bacterial tobacco wildfire and angular spot disease

Tobacco seeds (K326) were sown in nutrient soil, and seedlings with 2–4 euphylla were transplanted into individual pots. Plants at the six-leaf stage with a single central shoot were used for pathogen inoculation experiments. *Pseudomonas syringae* pv. *tabaci* (*Pst*) and *P. syringae* pv. *angulata* (*Psa*) strains were cultured overnight at 28℃ in LB liquid medium with shaking. The bacterial suspension was centrifuged at 5000 rpm for 10 min, washed with PBS, centrifuged again, and resuspended in PBS to a final concentration of OD_600_ = 0.001. Inoculation was performed by puncturing the leaves with a 1 mL syringe and injecting the pathogen suspension from the lower leaf surface. For each pathogenic strain, six to eight tobacco plants were inoculated, with two fully expanded leaves per plant inoculated at randomly selected positions. To evaluate the effect of TiO_2_ on the alleviation of disease symptoms, tobacco plants were sprayed with 9 ppb TiO_2_ 1 d before inoculation.

### 2.6. Inhibitory effect of TiO_2_ at different concentrations on fungi and bacteria

TiO_2_ was added to the PDA medium at concentrations of 72, 36, 18, and 9 ppb, and the fungi were transferred to a medium containing different TiO_2_ concentrations. The cultures were incubated at 28°C, and the colony diameters were photographed and analyzed to determine the growth rates of different fungi.

### 2.7. Extraction of plant nuclear protein/cytoplasmic protein

Nipponbare rice plants were cultured in MS liquid medium for two weeks, with 9 ppb TiO_2_ sprayed daily during the second week. Nuclear and cytoplasmic proteins were extracted using the Plant Nuclear Protein/Cytoplasmic Protein Extraction Kit (non-enzymatic method) (Solarbio, Cat: EX2232).

### 2.8. Measurement of wheat, soybean, and rice yields by TiO_2_ treatment

Soybean, wheat, and rice plants grown for two months were sprayed with a 9 ppb TiO_2_ solution twice a month until harvest. For wheat, measurements included plant height, spike length, weight per spike, number of grains per spike, and thousand-grain weight. For soybean, plant height, yield per plant, number of pods, total number of seeds, and hundred-grain weight were recorded. For rice, the tiller number, thousand-grain weight, yield per plant, and number of filled grains per panicle were measured.

### 2.9. Statistical analysis

The data were analyzed using GraphPad Prism 5.0. Student’s *t*-test was used for comparisons between two groups, whereas one-way analysis of variance (ANOVA) was performed for comparisons among multiple groups. Tukey’s multiple comparison test was used, and statistical significance was defined as *p* < 0.05.

## 3. Results

### 3.1. TiO_2_ promotes the growth of different crops

**Figure 1.**
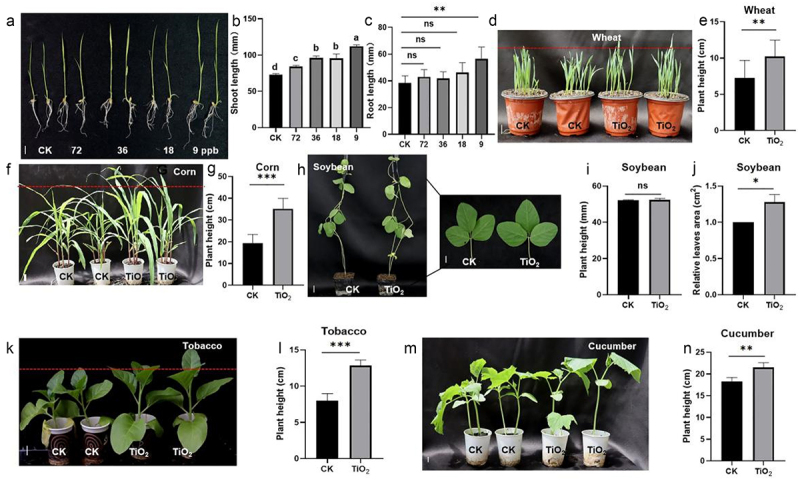
Growth-promoting effect of TiO_2_ on crops. (a) Rice seedlings were grown in liquid MS solution containing 72, 36, 18, and 9 ppb TiO_2_ for 7 d, respectively. (b) Shoot and (c) root lengths of the rice shown in (a). (d) TiO_2_ (9 ppb) was sprayed on the wheat to treat them. Seven-day-old wheat was photographed, and (e) the height of the wheat shown in (d) was calculated. (f) TiO_2_ (9 ppb) was sprayed on the corn. Seven-day-old corn was photographed, and (g) the height of the corn shown in (f) was calculated. (h) TiO_2_ (9 ppb) was sprayed on the soybean. Seven-day-old soybeans were photographed, and (i) the height of the soybeans shown in (g) and the relative leaf area (J) shown in (H) were calculated. (k) TiO_2_ (9 ppb) was sprayed on the tobacco. Fourteen-day-old tobacco plants were photographed and (l) the height of the tobacco shown in (k) was calculated. (m) TiO_2_ (9 ppb) was sprayed on the cucumber. Seven-day-old cucumbers were photographed, and (n) the height of the cucumbers shown in (M) was calculated. Data are presented as mean ± standard error (SE) (*n* > 10). Significant differences are denoted by asterisks (*p < 0.05, **p < 0.01, ***p < 0.001). ns: no significant difference. Scale bars = 1 cm.

To evaluate the concentration-dependent growth-promoting effects of TiO_2_, rice was grown in MS liquid medium with varying TiO_2_ concentrations. The results indicated that rice shoot length increased at TiO_2_ concentrations of 72, 36, 18, and 9 ppb, with the most significant growth promotion observed at 9 ppb (Figure 1(a,b)). However, root length was only promoted at 9 ppb TiO_2_ (Figure 1(c)). Based on these findings, 9 ppb TiO_2_ was selected for further investigation. Samples of wheat (Little Club), soybean (LiaoDou), tobacco (K326), cucumber (*Cucumis sativus*), and corn (B73) were treated with 9 ppb TiO_2_ NPs. TiO_2_ NPs significantly increased the plant height in wheat, tobacco, cucumber, and corn (Figure 1(d–g,k–n)). Although TiO_2_ NPs did not significantly affect soybean plant height, they notably increased the leaf size (Figure 1(h,j)).

### 3.2. The role of TiO_2_ in the nutrient uptake and transport mutations of rice

**Figure 2.**
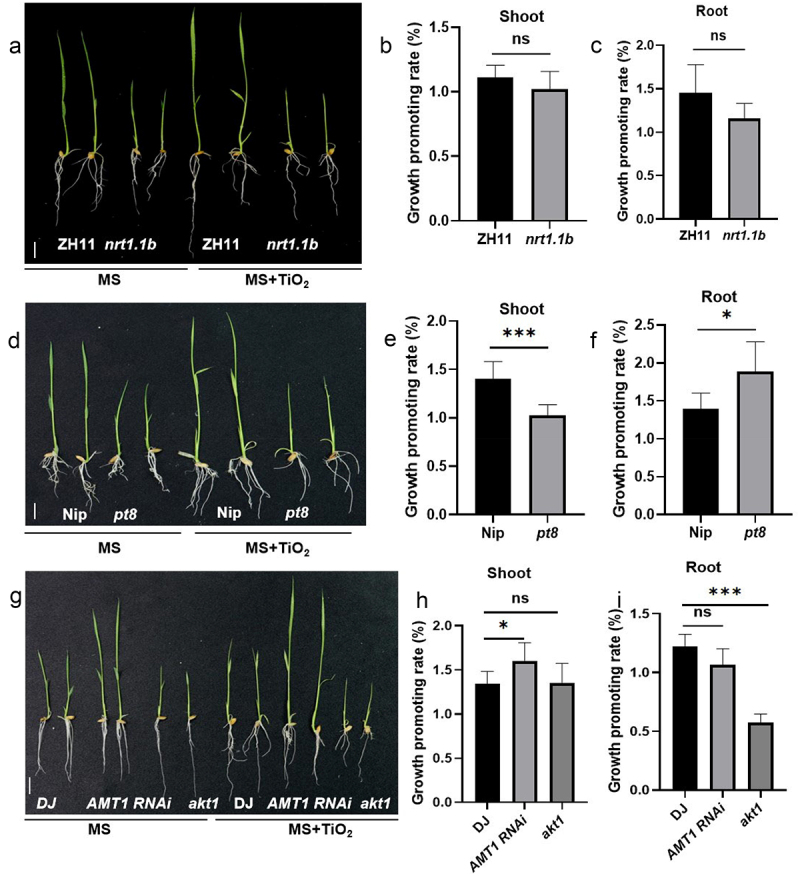
The promotion of rice growth by TiO_2_ is related to the absorption of nutrient elements. (a) ZH11 and *nrt1.1b* grown for 7 d in the liquid MS solution with or without 9 ppb TiO_2_ were photographed. (b) The growth-promotion rate of the rice shoots shown in (a) was calculated. (c) The growth-promotion rate of the roots in (a). (d) Morphology of Nip and *pt8* in MS nutrient solution containing 9 ppb TiO_2_ or without TiO_2_ for 7 d. (e) The growth-promotion rate of the shoots in (d). (f) The growth-promotion rate of the roots in (d). (g) Morphology of DJ, *AMT1 RNAi*, and *akt1* in MS nutrient solution containing 9 ppb TiO_2_ or without TiO_2_ for 7 d. (h) The growth-promotion rate of the shoots in (g). (i) The growth-promotion rate of the roots in (g). Data are presented as mean ± standard error (SE) (*n* > 10). Significant differences are denoted by asterisks (*p < 0.05, **p < 0.01, ***p < 0.001). ns: no significant difference. Scale bars = 1 cm.

In order to assess the correlation between nutrient uptake and TiO_2_-mediated growth promotion, we analyzed rice mutants including *nitrate transporter 1.1b* (*nrt1.1b*), *ammonium transporter 1* (*amt1*), *potassium channel* (*akt1*), and *phosphate transporter 8* (*pt8*), along with wild-type (WT) plants. These plants were cultured in liquid MS medium, with or without the addition of TiO_2_. The results showed that there was no significant difference in the growth-promoting efficiency of TiO_2_ on roots and shoots between the *nrt1.1b* rice mutant and the WT. Specifically, The *pt8* rice mutant exhibited shoot growth inhibition and root growth promotion. On shoots, no significant difference was observed between the *akt1* rice mutant and WT; however, root growth was significantly suppressed in *akt1* rice mutant. In contrast, *amt1* rice mutant showed no difference in root growth response to TiO_2_ compared to WT, while shoot growth was significantly promoted (Figure 2(a–i)).

### 3.3. TiO_2_ improves resistance disease of crops

**Figure 3.**
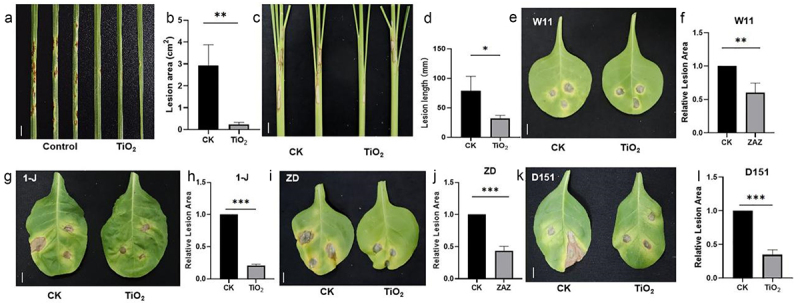
TiO_2_ improves disease resistance in plants. Plants were treated with 9 ppb TiO_2_ before inoculation. (a) Morphology of wheat leaves after inoculation with *Pgt*. (b) Lesion area of wheat shown in (a) was calculated. (c) Morphology of rice leaves after inoculation with *R. solani* AG1-IA. (d) Lesion length of the rice shown in (c) was calculated. (e) Morphology of tobacco leaves inoculated with *Pst* W11. (f) Relative lesion area of the tobacco shown in (e) was calculated. (g) Morphology of tobacco leaves after inoculation with *Psa 1-J*. (H) Relative lesion area of tobacco shown in (g) was calculated. (i) Morphology of tobacco leaves after inoculation with *Pst* ZD. (j) Relative lesion area of the tobacco shown in (i) was calculated. (k) Morphology of tobacco leaves inoculated with *Pst* D151. (l) Relative lesion area of tobacco shown in (k) was calculated. Data are presented as mean ± standard error (SE) (*n* > 8). Significant differences are denoted by asterisks (*p < 0.05, **p < 0.01, ***p < 0.001). ns: no significant difference. Scale bars = 1 cm.

Although TiO_2_ NPs significantly promote crop growth, the antagonistic relationship between growth and disease resistance balances the yield and disease resistance critical for crop development.^4,9,28^ To investigate the effects of TiO_2_ on disease resistance, the samples of wheat, rice, and tobacco were treated with TiO_2_ NPs after the inoculation with *Pgt*, *R. solani* and *Pst* and *Psa*, the pathogens responsible for the wheat stem rust, the rice ShB, and the tobacco wildfire and angular spot disease, respectively. The foliar application of TiO_2_ significantly enhanced wheat resistance to stem rust (Figure 3(a,b)), rice resistance to ShB (Figure 3(c,d)), and tobacco resistance to wildfire and angular spot disease (Figure 3(e–l)).

### 3.4. TiO_2_ inhibits the growth of pathogenic fungi and bacteria

**Figure 4.**
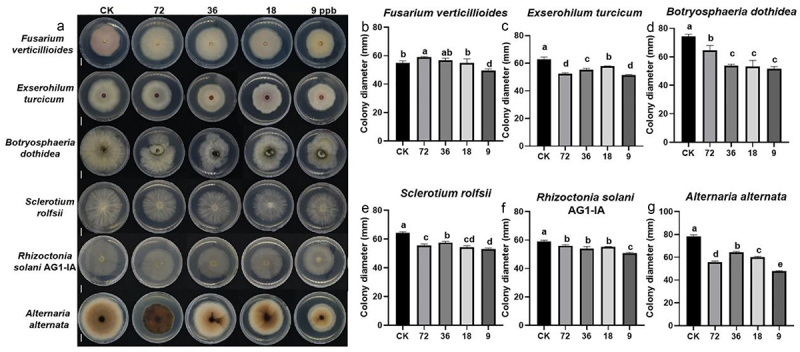
Antifungal activity of TiO_2_. (a) Cultures of different fungi grown on PDA plates treated with 9 ppb of TiO_2_. (b) Colony diameter of *Fusarium verticillioides* (a), (c) *Exserohilum turcicum*, (d) *Botryosphaeria dothidea*, (e) *Sclerotium rolfsii*, (f) *R. solani* AG1-IA, (G) *Alternaria alternata* shown in (a) were calculated. Different letters above the bars indicate significant differences (*p* < 0.05). Scale bars = 1 cm.

**Figure 5.**
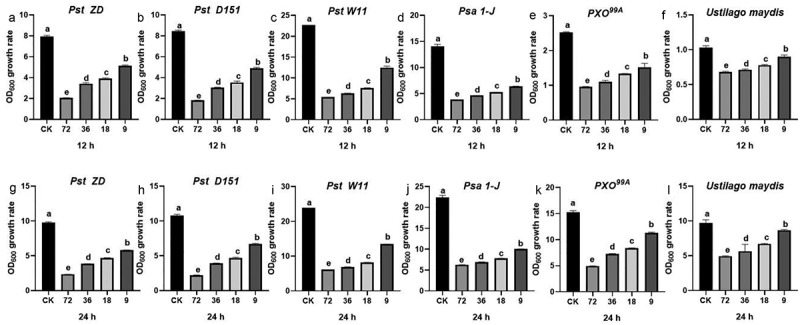
Antifungal and antibacterial activities of TiO_2_. (a) The OD_600_ growth rate of *Pst* ZD, (b) *Pst* D151, (c) *Pst* W11, (d) *Psa* 1-J, (e) PXO^99A^, and (f) *Ustilago maydis* was calculated after being inoculated with 72, 36, 18, and 9 ppb TiO_2_ for 12 h. (g) The OD_600_ growth rate of *Pst* ZD, (h) *Pst* D151, (i) *Pst* W11, (j) *Psa* 1-J, (k) PXO^99A^, and (l) *Ustilago maydis* was calculated after being inoculated with 72, 36, 18, and 9 ppb TiO_2_ for 24 h. Different letters above the bars indicate significant differences (*p* < 0.05).

TiO_2_ NPs significantly enhanced crop resistance to fungal and bacterial diseases, and their potential to directly inhibit pathogen growth was further investigated. The antifungal activity of TiO_2_ was tested against the fungal pathogens including *Fusarium verticillioides*, *Exserohilum turcicum*, *Botryosphaeria dothidea, Sclerotium rolfsii, R. solani* AG1-IA, *Alternaria alternata*, and U*stilago maydis*. The results revealed that TiO_2_ NPs at a concentration of 9 ppb slightly inhibited the growth of these fungi on the PDA medium (Figure 4(a–g), Table 1, and Figure 5(f,l)). The TiO_2_ application also suppressed the growth of bacterial pathogens, including *Pst* ZD, *Pst* D151, *Pst* W11, *Psa* 1-J, and PXO^99A^ (Figure 5(a–e,g–k)). These findings indicate that incorporating TiO_2_ NPs into the growth media effectively inhibits bacterial pathogen growth.

**Table 1.** Inhibitory rates of TiO_2_ on different fungi.

Fungi	Concentration (ppb)	Inhibitory rate (%)
*Exserohilum turcicum*	72	19.1373404
	36	13.6835606
	18	9.1960079
	9	20.9044331
*Botryosphaeria dothidea*	72	14.6899402
	36	30.7471218
	18	31.7836538
	9	33.952015
*Fusarium verticillioides*	72	−8.6496515
	36	−4.2884684
	18	−0.4612958
	9	11.2697936
*Sclerotium rolfsii*	72	15.1675881
	36	11.6400688
	18	17.6742399
	9	19.515355
*Rhizoctonia solani* AG1-IA	72	13.7605068
	36	12.5301856
	18	7.19657149
	9	5.6996032
*Alternaria alternata*	72	31.876747
	36	19.7620466
	18	25.9948556
	9	43.2714806

### 3.5. TiO_2_ improves rice growth and disease resistance by regulating chlorophyll biosynthesis and light signaling

**Figure 6.**
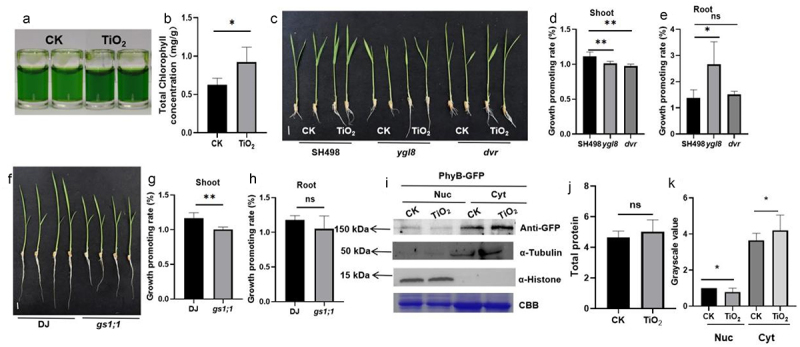
TiO_2_ improves the chlorophyll content of rice and inhibits PhyB translocation from the cytosol to the nucleus. (a) Chlorophyll content was measured after 14 d of 9 ppb TiO_2_ treatment. Fig. 6 TiO_2_ improves the chlorophyll content of rice and inhibits PhyB translocation from the cytosol to the nucleus. (a) Chlorophyll content was measured after 14 d of treatment with 9 ppb TiO_2_. (b) Total chlorophyll content of rice grown in MS liquid medium containing 9 ppb TiO_2_ for 14 d. (c) Seven-day-old SH498, *dvr*, and *ygl8* seedlings were grown in MS solution with or without 9 ppb TiO_2_. (d) Growth-promotion rate of shoots in (c). (e) Growth-promotion rate of roots in (c). (f) Seven-day-old germinated DJ and *gs1;1* seedlings were transferred to MS nutrient solution with or without 9 ppb TiO_2_. (g) Growth-promotion rate of shoots in (f). (h) Growth-promotion rate of roots in (f). (i) Distribution of PhyB protein in rice *PhyB-OX* treated with 9 ppb TiO_2_ for 7 d. (j) Total protein content. (k) Grayscale value of (l). Data are presented as mean ± standard error (SE) (*n* > 8). Significant differences are denoted by asterisks (*p < 0.05, **p < 0.01, ***p < 0.001). ns: no significant difference.

TiO_2_ NPs not only enhance rice growth but also improve resistance to ShB. The mechanism underlying this dual effect was investigated. During the growth promotion experiment, rice leaves treated with TiO_2_ NPs displayed a significantly darker green coloration (Figure 6(a)), with a notable increase in chlorophyll content (Figure 6(b)). To explore the association between TiO_2_-induced growth and chlorophyll content, the rice chlorophyll biosynthetic gene mutants *dvr* and *ygl8*, as well as the *gs1;1* mutant, were analyzed, with glutamate serving as the chlorophyll precursor. The results showed that after TiO_2_ treatment, *dvr* and *ygl8* rice mutants exhibited shoot growth inhibition. TiO_2_ significantly promoted root growth in the wild-type, *ygl8*, and *dvr* plants, with a much higher promotion rate observed in the *ygl8* plants than in the wild-type and *dvr* plants (Figure 6(c–e)). *GS1;1* is the glutamine synthase gene, which is the first step of ammonium assimilation. Next, the glutamine synthase gene mutant *gs1;1* was selected as the research object to investigate the role of ammonium assimilation in promoting the growth process of rice. The results demonstrate that inhibition of both shoot and root growth was observed in the *gs1;1* rice mutant (Figure 6(f–h)).

The role of TiO_2_ NPs in light signaling was investigated, focusing on Phytochrome B (PhyB) as a key red and far-red photoreceptor with conserved functions in monocots and dicots. Previous studies have shown that PhyB regulates rice growth, nutrient uptake, and disease resistance.^29–31^ Although TiO_2_ NPs are known to significantly enhance photosynthetic efficiency,^32^ their role in light signaling remains unclear. To address this, PhyB-GFP plants were treated with TiO_2_ NPs, and cytosolic and nuclear proteins were extracted. Western blot analysis revealed that TiO_2_ NPs treatment significantly reduced the PhyB-GFP levels in the nucleus while increasing its levels in the cytosol compared to the control, indicating that TiO_2_ NPs inhibited the translocation of PhyB from the cytosol to the nucleus (Figure 6(i–k)). These findings suggest that TiO_2_ NPs enhance photosynthesis while slightly inhibiting PhyB-mediated light signaling.

### 3.6. TiO_2_ increases the yield of wheat and soybean

**Figure 7.**
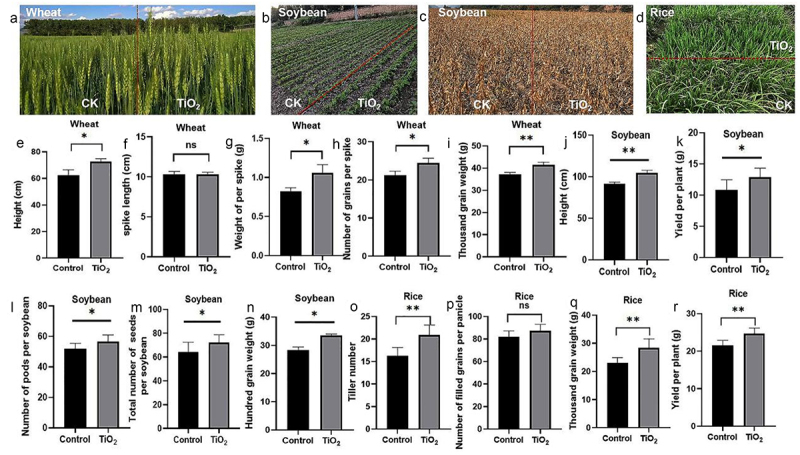
TiO_2_ increases rice, soybean, and wheat yields. (a)–(d) Field-grown wheat, soybean, and rice following TiO_2_ treatment were photographed. (e) Height of wheat, (f) spike length, (g) weight per spike, (h) number per spike, and (i) thousand-grain weight of wheat after TiO_2_ treatment. (j) Height of soybean, (k) yield per soybean plant, (l) number of pods per soybean, (m) total number of seeds per soybean, and (n) hundred grain weight of soybean after TiO_2_ treatment. (o) Tiller number, (p) number of filled grains per panicle, (q) thousand-grain weight number of filled grains per panicle, and (R) yield per plant of rice after TiO_2_ treatment. Data are presented as mean ± standard error (SE). Significant differences are denoted by asterisks (*p < 0.05, **p < 0.01). ns: no significant difference.

The application of TiO_2_ NPs can promote rice seedling growth by activating chlorophyll biosynthesis to enhance photosynthesis and boost the production yield. To evaluate the effects of TiO_2_ NPs on wheat, soybean, and rice yields, field tests were conducted by applying TiO_2_ to two-month-old plants twice a month until harvest. The results showed that while TiO_2_ NPs did not affect wheat spike length, they increased plant height, weight per spike, and thousand-grain weight (Figure 7(a,e–i)). In soybeans, the TiO_2_ NPs treatment improved plant height, yield per plant, number of pods, total seed number, and hundred-grain weight (Figure 7(b,c,j–n)). For rice, TiO_2_ NPs enhanced tiller number, thousand-grain weight, and yield per plant, although it did not affect the number of filled grains per panicle (Figure 7(d,o–r)). These findings demonstrate that TiO_2_ NPs can enhance the grain weight and improve the overall crop yield of wheat, soybean, and rice.

## 4. Discussion

Ensuring food security relies on improving both output and grain production capacity. As a future development direction, nano-agriculture represents an interdisciplinary innovation aligned with Agriculture 4.0, embodying the enhanced quality and productivity in the agricultural sector.^33^ TiO_2_ can increase the content of chlorophyll, thereby enhancing photosynthesis and promoting plant growth. On the other hand, TiO_2_ can directly inhibit the growth of pathogenic fungi and bacteria, thereby promoting plant disease resistance. Therefore, TiO_2_ can balance the relationship between crop yield and disease resistance. It can not only promote crop growth but also increase the yield of various crops. This study analyzed the role of TiO_2_ NPs in promoting plant growth, enhancing yield, and improving disease resistance, highlighting their significant potential for agricultural applications.

### 4.1. TiO_2_ NPs promotes plant growth

TiO_2_ NPs have been reported to promote plant growth.^34^ However, the underlying mechanism remains unclear. To investigate their function, the optimal concentration of TiO_2_ NPs for plant growth was tested in rice. Although shoot growth was promoted across a range of TiO_2_ concentrations, root growth was enhanced only at higher concentrations, suggesting differential regulatory mechanisms for shoots and roots. The optimal concentration (9 ppb) identified in rice also promoted the growth of wheat, soybean, tobacco, cucumber, and corn, confirming the growth-promoting effects of TiO_2_ NPs. To explore how TiO_2_ could promote growth, the relationship between nutrient uptake and TiO_2_-induced growth was analyzed using a genetic approach. After treatment with TiO_2_, the rice mutants *nrt1.1b*, *akt1* and *pt8* exhibited shoot growth inhibition, while the *amt1* rice mutant did not exhibit shoot growth inhibition. The results indicated that TiO_2_ NPs can enhance nitrate, potassium, and phosphate uptake to stimulate shoot growth. The *nrt1.1b*, *akt1*, and *amt1* rice mutants but not *pt8* exhibited root growth inhibition, suggesting that the promotion of rice growth by TiO_2_ is related to the pathways of nitrate, potassium, and ammonium uptake, further highlighting a distinction between TiO_2_ NP-induced growth mechanisms in shoots and roots. Because nitrogen uptake can play a critical role in TiO_2_ NP-induced seedling growth, nitrogen metabolism was analyzed. Glutamine synthetase (GS), which is an enzyme that incorporates ammonium into glutamine,^35^ was observed to be essential. This was evident from the phenotype of plants with the *gs1;1* mutation, which is regarded as a mutation affecting a key cytosolic GS isoform.^36^ These mutant plants displayed a marked reduction in glutamine synthesis and a subsequent negative impact on plant growth and development, strongly indicating that GS plays a vital role in nitrogen metabolism and overall plant physiological processes. Amino acids are key metabolites in nitrogen metabolism that contribute to chlorophyll production.^37^ As anticipated, TiO_2_ NP treatment significantly increased the chlorophyll content, serving as a key component of photosynthesis to directly influence the growth rate and yield of crops. Higher photosynthetic efficiency can lead to greater organic matter accumulation, promoting crop growth and development and enhancing resistance to environmental stresses, such as drought, diseases, and insect attacks. The adaptability of crops to various environmental challenges can be strengthened by improving their photosynthetic efficiency.^38^ Analysis of the rice chlorophyll biosynthetic gene mutants, *ygl8* and *dvr*, revealed that TiO_2_ NP-induced shoot growth was inhibited in these rice mutants, whereas root growth was unaffected in the *dvr* mutant and even enhanced in the *ygl8* mutant. These findings suggest that TiO_2_ NPs promote nitrogen uptake, metabolism, and photosynthesis to drive shoot growth, while root growth is regulated differently, as the root tissues may lack chloroplasts and rely on amino acid biosynthesis for TiO_2_ NP-induced promotion. Further research is required to explore the molecular mechanisms underlying TiO_2_ NP-induced root growth.

TiO_2_ NPs affects plant growth by having a significant regulatory effect on plant nitrogen metabolism. Under the action of ultraviolet rays, TiO_2_ NPs can convert nitrogen oxides in the atmosphere into NO_3_^−^. Meanwhile, TiO_2_ NPs can also significantly affect the activities of various key enzymes in the nitrogen metabolism pathway, including GS, nitrite reductase (NiR), glutamate dehydrogenase (GDH), and alanine aminotransferase (GPT). These enzymes jointly participate in the nitrogen assimilation metabolic network, cooperatively regulating the conversion process from inorganic nitrogen to organic nitrogen, ultimately promoting the assimilation of inorganic nitrogen into nitrogen-containing organic substances such as chlorophyll and amino acids, and then participating in the biosynthesis of proteins.^39^ The above regulatory effects may promote plant growth. In addition, TiO_2_ nanomaterials have excellent photocatalytic properties, which can improve the utilization efficiency of light energy by plants and promote the progress of photosynthesis. After TiO_2_ NPs of appropriate concentration enters the chloroplast, by promoting the transfer of energy electrons in the photosynthetic electron transport chain, NADP^+^ is reduced to NADPH and coupled with photophosphorylation, thereby converting electron energy into ATP and accelerating the photolysis of water and the precipitation of O_2_.^40^ It alleviated the chloroplast destruction and chlorophyll degradation in leaf tissues.^41^ Therefore, TiO_2_ NPs can promote crop growth by regulating nitrogen metabolism and photosynthesis.

### 4.2. TiO_2_ NPs promotes crop yield

Since TiO_2_ NPs can promote plant growth, their role in yield production was investigated by treating 2-month-old wheat, soybean, and rice with TiO_2_ NPs twice a month until harvest, followed by evaluation of crop traits and yields. The results showed that TiO_2_ NPs increased seed weight across all three crops, as confirmed by the 1000- or 100-grain weight. However, TiO_2_ NPs exhibited crop-specific effects. They increased the height of wheat and soybean but not that of rice and increased the tiller number in rice. TiO_2_ NPs increased the number of soybean pods but not that of filled grains per panicle in rice. These differences may be linked to the growth conditions and physiological characteristics of each crop, where wheat was field-grown, rice was cultivated in paddy soil, and soybean was a field-grown legume with self-nitrogen fixation. TiO_2_ NPs enhanced nitrogen uptake, with wheat and soybean primarily utilizing nitrate and rice relying on ammonium, which may explain their varied responses. TiO_2_ NPs consistently increased the seed weight, likely by enhancing photosynthesis to produce more carbohydrates. Combined with improved nitrogen uptake and assimilation, TiO_2_ NPs likely increased the starch and amino acid content in seeds, contributing to the higher seed weight in these crops. It has been reported that TiO_2_ NPs can increase the chlorophyll content of pakchoi, thereby enhancing photosynthesis and ultimately increasing the yield of pakchoi.^24^ In addition, TiO_2_ NPs can alleviate the growth inhibition induced by polystyrene nanoplastics by regulating the carbon and nitrogen metabolism of corn through the melatonin signaling pathway. Corn seedlings treated with Nano-TiO_2_ exhibit stronger capabilities in photosynthesis, sucrose synthesis, nitrogen assimilation and protein synthesis. Meanwhile, by regulating the antioxidant system, the treatment with Nano-TiO_2_ alleviates the oxidative damage of corn seedlings.^39^ Therefore, TiO_2_ NPs play an extremely important role in increasing the yields of crops and pakchoi. Since the 21st century, the development and application of nanomaterials have been increasingly rapid and extensive. As the nano-metal oxide with the largest annual output globally, TiO_2_ NPs may also have certain impacts on the environment while being widely and long term applied.^42^ However, this kind of influence is a long-term process. Therefore, it is of great significance to assess the environmental impact of using TiO_2_ NPs in the future, and to provide a scientific basis for the sustainable application of NPs in the fields of agriculture and the environment.

### 4.3. Multiple functions of TiO_2_ NPs in crop disease protection

The relationship between crop yield and disease resistance is often antagonistic, with high yields typically being associated with reduced disease resistance. TiO_2_ NPs treatment significantly improved wheat resistance to stem rust, rice to ShB, and tobacco to wildfire and angular spot disease, suggesting that TiO_2_ NPs simultaneously enhanced plant yield and disease resistance. Several tests were conducted to investigate this dual effect. Direct *in vitro* inhibition of pathogenic bacteria and fungi by TiO_2_ NPs was initially tested, revealing that TiO_2_ NPs slightly inhibited bacterial and fungal growth. However, this inhibition may be insufficient for effective disease control, suggesting that TiO_2_ NPs may regulate other signaling pathways to achieve broad-spectrum resistance in plants. In addition, TiO_2_ NPs can adhere to the surface of plants to form a protective film. This film can prevent the direct contact between pathogenic bacteria and plant cells, reducing the infection opportunities of pathogenic bacteria. For example, after spraying TiO_2_ NPs on cucumber plants, it was found that they had a significant blocking effect on the invasion of downy mildew bacteria in cucumbers.^43^ It is reported that TiO_2_ NPs achieve an efficient sterilization effect by agglomerating and coating microorganisms and inducing them to produce reactive oxygen species (ROS), thereby destroying the cell membrane and organelle structure of microorganisms.^44^ Genetic analysis using nutrient transporter rice mutants revealed that TiO_2_ NPs enhance K^+^, Pi, and NO_3_^−^ uptake to promote plant growth. Previous research has demonstrated that *nrt1.1b* rice mutants are more susceptible, whereas *akt1* and *pt8* rice mutants are less susceptible to ShB in rice, suggesting that the mechanism underlying TiO_2_ NP-mediated ShB resistance is complex.^45^ Furthermore, the *nrt1.1b* mutant’s increased susceptibility to ShB, along with the *gs1;1* mutant’s heightened susceptibility to ShB, blast, and bacterial blight,^45^ highlights the critical role of nitrogen assimilation in TiO_2_ NP-mediated broad-spectrum resistance.

The role of TiO_2_ NPs in chlorophyll biosynthesis was analyzed to determine how glutamine synthetase regulates rice resistance. TiO_2_ NPs increased the chlorophyll content in rice, and the shoot growth-promoting effect of TiO_2_ NPs was eliminated in the rice chlorophyll mutants *ygl8* and *dvr*, indicating that chlorophyll synthesis is a key step in TiO_2_ NP-mediated growth promotion. Additionally, the *ygl8* and *dvr* rice mutants were more susceptible to rice ShB but not to blast or bacterial blight,^46,47^ suggesting that the TiO_2_ NP-induced activation of chlorophyll biosynthesis primarily contributes to ShB resistance. Since chlorophyll biosynthesis is closely linked to photosynthesis, and TiO_2_ NPs have been shown to accelerate photosynthesis rates,^34^ their role in light signaling was tested. Light signaling can regulate nutrient acquisition and utilization,^48^ and phytochromes (Phys), particularly PhyB, act as red-light photoreceptors involved in nitrogen transport.^49,50^ PhyB can be activated by light via conversion of PhyB-pr to PhyB-pfr, triggering nucleation. TiO_2_ NPs treatment did not affect the total PhyB protein levels but slightly inhibited PhyB nucleation, suggesting that TiO_2_ NPs suppressed PhyB-dependent light signaling. Notably, PhyB negatively regulates rice resistance to ShB, while positively regulating resistance to blast,^31,51^ highlighting the complexity of PhyB signaling. In conclusion, TiO_2_ NPs protected rice from ShB by inhibiting fungal growth, suppressing PhyB nucleation, and enhancing nitrate uptake and assimilation. Furthermore, TiO_2_ NP-mediated broad-spectrum disease control likely involved fungal and bacterial growth inhibition and activation of nitrogen assimilation.

**Figure 8.**
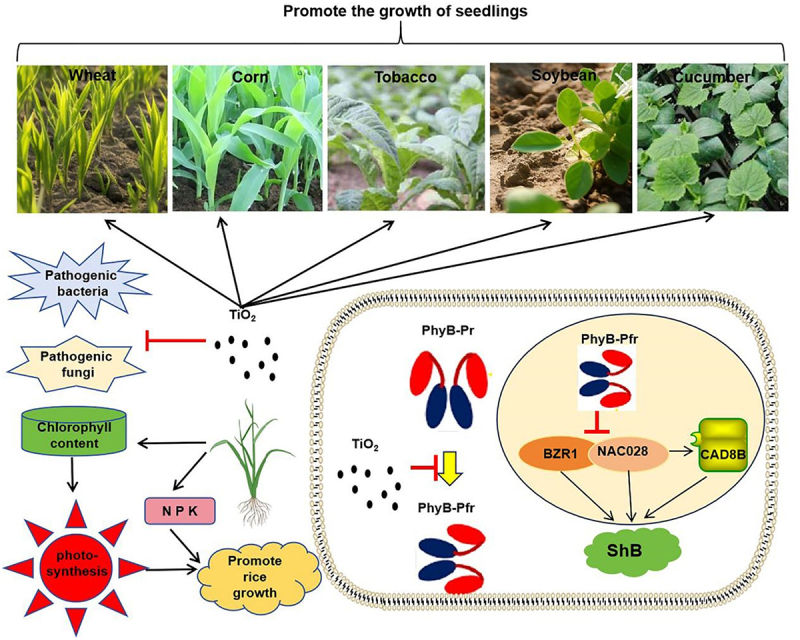
Working model of TiO_2_ in promoting plant growth and resistance: TiO_2_ inhibits the nuclear import of PhyB and promotes the resistance of rice to ShB. TiO_2_ mediates the activation of nitrogen, phosphorus, and potassium absorption as well as photosynthesis to enhance plant growth. TiO_2_ promotes the growth of wheat, corn, tobacco, soybeans and cucumbers at the seedling stage. TiO_2_ directly inhibits growth of pathogenic fungi and bacteria. Furthermore, TiO_2_ inhibits PhyB translocation from the cytosol to the nucleus, thereby enhancing the resistance of rice to ShB.

In recent years, the biological effects of TiO_2_ NPs have become a major focus of environmental science. This study revealed the mechanisms by which TiO_2_ NPs promoted rice growth and enhanced resistance to ShB via nutrient pathways, photosynthesis, and PhyB-mediated light signaling. TiO_2_ NPs enhanced energy accumulation during photosynthesis, thereby promoting rice growth (Figure 8). These findings provide valuable insights into the potential applications and risk assessment of NPs in agricultural production.

## Data Availability

Data will be made available on request.
